# 3T MRI evaluation of regional catecholamine-producing tumor-induced myocardial injury

**DOI:** 10.1530/EC-18-0553

**Published:** 2019-03-25

**Authors:** Satoshi Higuchi, Hideki Ota, Takuya Ueda, Yuta Tezuka, Kei Omata, Yoshikiyo Ono, Ryo Morimoto, Masataka Kudo, Fumitoshi Satoh, Kei Takase

**Affiliations:** 1 Department of Diagnostic Radiology, Tohoku University Hospital, Sendai, Miyagi, Japan; 2 Division of Nephrology, Endocrinology and Vascular Medicine, Department of Medicine, Tohoku University Hospital, Sendai, Miyagi, Japan; 3 Division of Clinical Hypertension, Endocrinology and Metabolism, Tohoku University Graduate School of Medicine, Sendai, Miyagi, Japan

**Keywords:** catecholamine-induced myocardial injury, myocardial strain, myocardial T1-mapping, regional difference

## Abstract

**Objective:**

Regional differences in cardiac magnetic resonance, which can reveal catecholamine-induced myocardial injury in patients with pheochromocytoma, have not yet been assessed using 3T magnetic resonance imaging. We evaluated these differences using myocardial T1-mapping and strain analysis.

**Design and Methods:**

We retrospectively reviewed 16 patients newly diagnosed with catecholamine-producing tumors (CPT group) and 16 patients with essential hypertension (EH group), who underwent cardiac magnetic resonance imaging between May 2016 and March 2018. We acquired 3T magnetic resonance cine and native T1-mapping images and performed feature-tracking-based strain analysis in the former.

**Results:**

Global cardiac function, morphology, global strain and peak strain rate were similar, but end-diastolic wall thickness differed between groups (CPT vs EH: 10.5 ± 1.7 vs 12.6 ± 2.8 mm; *P* < 0.05). Basal, but not apical, circumferential strain was significantly higher in the CPT than the EH group (19.4 ± 3.2 vs 16.8 ± 3.6 %; *P* < 0.05). Native T1 values were significantly higher in CPT than in EH patients, in both the basal septum (1307 ± 48 vs 1241 ± 45 ms; *P* < 0.01) and the apical septum (1377 ± 59 vs 1265 ± 58 ms; *P* < 0.01) mid-walls. In the CPT, but not in the EH group, native T1 values in the apical wall were significantly higher than those in the basal wall (*P* < 0.01).

**Conclusion:**

3T magnetic resonance-based T1-mapping can sensitively detect subclinical catecholamine-induced myocardial injury; the influence of catecholamines may be greater in the apical than in the basal wall.

## Introduction

Pheochromocytomas and paragangliomas are catecholamine-producing tumors (CPTs). The prevalence of pheochromocytoma in patients with hypertension in general outpatient clinics varies between 0.2 and 0.6% (1). Excess catecholamines are secreted from the adrenal gland or extra-adrenal chromaffin cells in CPTs, spontaneously. Patients with these tumors may present with various clinical symptoms, such as episodic headaches, sweating, tachycardia, palpitations and paroxysmal hypertension (2). The increase in catecholamine production can also lead to high morbidity and mortality, due to catastrophic cardiovascular complications, including myocardial ischemia, aortic dissection, stroke, hypertensive crisis and peripheral ischemia (3, 4, 5, 6).

The excess catecholamines and their metabolites have direct toxic as well as receptor-mediated effects on myocardial tissue (7, 8). Progressive cardiac injury leads to cardiac remodeling and decreased cardiac function, independent of the hypertensive effect, and results in catecholamine-induced cardiomyopathy (9). The prevalence of catecholamine-induced cardiomyopathy in patients with pheochromocytoma is 10–11% (10, 11). Patients with catecholamine-induced cardiomyopathy often have a poor prognosis due to fatal arrhythmias, heart failure and circulatory collapse (11, 12). However, patients without cardiac remodeling and dysfunction can also have catecholamine-induced cardiac damage. Previous reports have indicated that the sudden release of catecholamines from the pheochromocytoma is associated with QT prolongation and ventricular tachycardia (VT); torsades de pointes or VT have been observed even in young pheochromocytoma patients without cardiac dysfunction (13, 14, 15).

Cardiac magnetic resonance (CMR) imaging is a valuable tool for non-invasive assessment of cardiac morphology and function, and characterization of myocardial tissue. Cine magnetic resonance (MR) images allow evaluation of left ventricular volumetric parameters as well as wall motion velocity, myocardial strain and strain rate, which can facilitate detection of local or global dysfunction using wall motion tracking methods (16). In terms of tissue characterization, myocardial T1-mapping provides myocardial longitudinal (spin-lattice) relaxation times reflecting myocardial tissue properties, such as edema, fibrosis or fat infiltration, without the use of contrast medium. This technique is a sensitive tool for detecting local and diffuse myocardial pathophysiological changes (17).

A recent report has shown that patients with pheochromocytoma had a lower left ventricular ejection fraction, lower peak systolic circumferential strain, lower diastolic strain rate and higher myocardial native T1 values than healthy controls and patients with hypertension, based on the results obtained using a 1.5T MR scanner (18). However, there have been no reports of 3T CMR imaging providing higher signal-to-noise ratio than 1.5T CMR imaging in CPT patients. The previous 1.5T MR study used myocardial tagging to obtain the radial and circumferential strains and did not show the longitudinal strain.

Furthermore, the left ventricle contains apical–basal gradients of β adrenoreceptors (βARs) (which are responsible for increased responsiveness to epinephrine) and sympathetic innervation, with the apex characterized by the highest βAR concentration (19). There is a hypothesis that the distribution of βARs contributes to abnormal systolic wall motion in Takotsubo cardiomyopathy (TC), where hypokinetic apical wall with ballooning and hyperkinetic basal wall are observed (20). A recent study reported that acute Takotsubo-like cardiomyopathy was found in up to 3% of patients with functional pheochromocytoma and paraganglioma (21), whereas only 0.02% of all hospitalized patients suffered from TC (22). However, the regional distribution of the degree of catecholamine-induced myocardial damage has not been evaluated in patients with CPTs.

We hypothesized that CMR may be able to identify the regional variance of the catecholamine-induced myocardial damage in patients with CPTs without contrast medium. We aimed to evaluate whether CMR could detect subclinical catecholamine-induced cardiac injury in patients with CPTs, as compared to patients with essential hypertension (EH), using a 3T MR scanner. We also evaluated whether there were differences in the degree of myocardial injury between the basal and apical regions using myocardial T1-mapping and wall motion tracking.

## Materials and methods

### Ethics

This retrospective study was approved by the Ethics Committee of Tohoku University School of Medicine (#2018-1-133). The requirement for obtaining written informed consent from the patients was waived by the ethics committee.

### Patients

Sixteen consecutive patients with newly diagnosed CPT, who underwent CMR examinations at our institution from March 2016 to May 2018 and who did not meet the exclusion criteria, were included in the study. The presence of CPT was diagnosed by endocrinologists based on serum and urinary biochemical tests, ^123^I metaiodobenzylguanidine scintigraphy and MR imaging. Another 16 consecutive patients with EH, who underwent CMR imaging within the same period of time, were also included. These patients were referred to our institution because of refractory hypertension, but were diagnosed with EH by the same endocrinologists after ruling out secondary causes of hypertension. Exclusion criteria were a history of cardiac events (heart failure, myocardial infarction, valvular disease and arrhythmias) and severe renal dysfunction (estimated glomerular filtration rate <30 mL/min).

Demographic characteristics of the patients were collected; these included sex, age, BMI, body surface area, family history of EH, medical history of diabetes mellitus, smoking history, number of antihypertensive drugs, systolic blood pressure (measured at home) and disease duration (the interval from the appearance of subjective symptoms or detection of hypertension to the diagnosis). In the CPT group, 24-h urinary metanephrine and normetanephrine levels were evaluated to determine the biochemical phenotype (adrenergic or noradrenergic). Patients are classified as adrenergic if the increment of metanephrine exceeded 5% of the combined metanephrine and normetanephrine increments. Patients in whom these criteria were not fulfilled and in whom normetanephrine levels exceeded the upper limits of normal were classified as noradrenergic (23).

### CMR protocol

All patients were imaged with a 3T whole body MR scanner (MAGNETOM Trio A Tim System; Siemens Healthineers) before surgery. Scanning protocols included cine and precontrast T1 maps. Native T1 maps were acquired using a modified Look-Locker inversion recovery (MOLLI) technique. Cine images were acquired at 20 frames per slice per beat in short-axis, long-axis and four-chamber views. Details of the parameters in cine and T1-mapping are provided in Supplementary Table 1 (see section on supplementary data given at the end of this article). To measure the longer T1 time accurately, independent of patient heart rate for native T1 values, we applied the (8(2)2) scheme indicating two inversion pulses with acquisition of images for eight heart beats, followed by a recovery of two heart beats and a second inversion pulse with images acquired for two heart beats. In-plane motion correction was performed and T1 maps were automatically generated.

### Image analysis

We analyzed MR images using a workstation (Ziostation2; Ziosoft, Tokyo, Japan). We measured the end-systolic and end-diastolic ventricular septal wall thickness; left ventricle ejection fraction (LVEF); the end-diastolic, end-systolic and stroke volume index (EDVI, ESVI and SI, respectively); and the cardiac index (CI) from short-axis cine images. The left ventricular myocardial mass index (LVMI) was also acquired from short-axis cine images, using Simpson’s technique. We analyzed myocardial strain and strain rate using the MR Wall Motion Tracking application (Vitrea, Canon Medical Systems, Otawara, Japan). Radial strain and circumferential strain were derived from the short-axis images and longitudinal strain was derived from the two- and four-chamber images. We used the average of the longitudinal strain from the two- and four-chamber images as the global longitudinal strain. We also evaluated basal and apical wall strain from segmented strain data.

**Figure 1:**
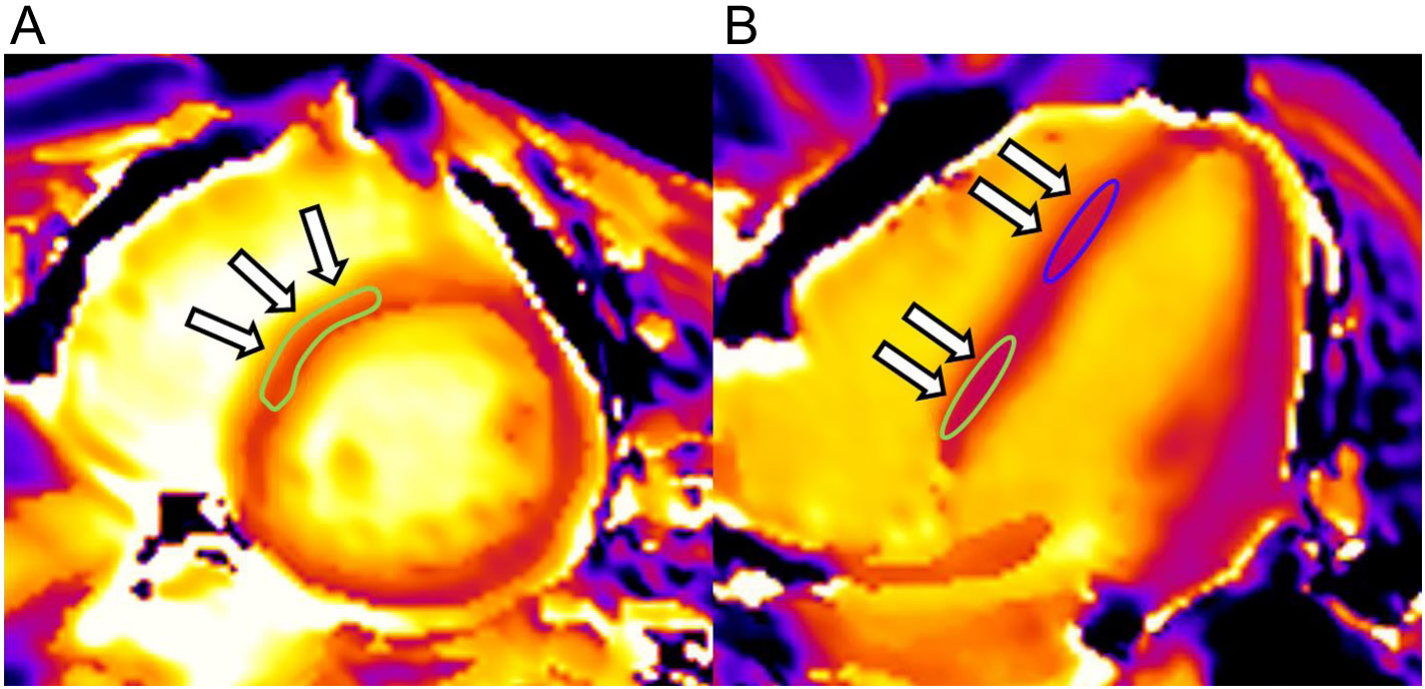
Images from a 49-year-old male with pheochromocytoma. Sample of region of interest for evaluation of native T1 values in the basal septum (A and B) and apical septum (B). Arrows indicate the region of interest for native T1 measurement.

Two radiologists (H O and S H, with 16 and 5 years of experience, respectively) independently measured native T1 values, for analysis of interobserver reproducibility. Regions of interest for evaluation of native T1 values were placed on the mid-wall of the basal septum and the mid-wall of the apical septum, using the short-axis and four-chamber T1 images (Fig. 1). T1 values measured by a single reviewer were considered to be representative of individual segments. Reviewers were blinded to the patients’ demographics.

### Statistical analysis

Descriptive statistics are presented as means and standard deviations (s.d.) for continuous variables and as the number of cases and percentages per group for categorical variables. Nonparametric data are shown as medians with interquartile ranges (IQRs). The normality of MR parameters and patient demographic data were tested using the Kolmogorov–Smirnov test. Variables between CPT and EH groups were compared using Student’s *t*-test or the Mann–Whitney *U* test. For dichotomous data, Fisher’s exact test was used to analyze the difference of proportions between the groups. We analyzed paired data using the paired *t*-test or the Wilcoxon signed-rank test. Interobserver reproducibility of native T1 values was evaluated using intraclass correlation coefficients (ICCs). Based on the 95% confident intervals (CI) of the ICCs, values less than 0.5, values 0.5–0.75, 0.75–0.9 and those greater than 0.9 were considered indicative of poor, moderate, good and excellent reliability, respectively. Statistical analyses were performed using SPSS version 21.0 (IBM). *P* < 0.05 was considered statistically significant.

## Results

### Patient characteristics

Among the included 16 patients with CPT, 15 underwent surgical resection; histopathological examinations revealed pheochromocytoma in 13 patients, paraganglioma in 1 patient and ganglioneuroblastoma in 1 patient. The other patient had a mediastinal tumor that was clinically diagnosed as a paraganglioma after positive biochemical tests and scintigraphy findings; this patient did not undergo surgery, as the patient experienced aortic dissection during the presurgical period. The baseline patient characteristics are summarized in Table 1.

**Table 1:** Patient demographic characteristics.

	CPT group (*n* = 16)	EH group (*n* = 16)	*P* value
Age (years)	52 ± 13	53 ± 14	0.87
Female (% (n))	62 (10/16)	50 (8/16)	0.72
BMI (kg/m^2^)*	22.6 ± 3.2	25.8 ± 3.8	**0.02**
BSA (m^2^)	1.6 (1.6–1.7)	1.7 (1.5–1.8)	0.62
Family history of hypertension (% (*n*))	75 (12/16)	88 (14/16)	0.65
DM (% (*n*))	19 (3/16)	13 (2/16)	1.00
Smoking history (% (*n*))	50 (8/16)	69 (11/16)	0.47
Number of antihypertensive drugs (n)	2 (0–2)	2 (1–2)	0.64
Home sBP (mmHg)	130 (116–150)	130 (120–148)	0.90
Disease duration (y)*	2.3 (1–5.5)	6.5 (2.3–16.8)	**0.02**

BMI, body mass index; BSA, body surface area; CPT, catecholamine-producing tumor; DM, diabetes mellitus; EH, essential hypertension; sBP, systolic blood pressure. Bold indicates statistical significance.

None of the patients from the EH group were excluded from the analysis. The EH group demonstrated a significantly larger BMI (25.3 ± 3.8 vs 22.6 ± 3.2 kg/m^2^; *P* = 0.02) and longer disease duration than the CPT group (6.5 (IQR, 2.3–16.8) vs 2.3 (IQR, 1–5.5) years; *P* = 0.02). There were no significant differences between the two groups with regard to the other demographic parameters.

### Left ventricular morphology and function

Left ventricular (LV) end-diastolic walls were significantly thicker in the EH group than in the CPT group (12.6 ± 2.8 mm vs 10.5 ± 1.7 mm; *P* = 0.025). Other LV functional parameters (end-systolic wall thickness, LVEF, EDVI, ESVI, SI, CI, LVMI) demonstrated no significant differences between the two groups (Table 2).

**Table 2:** Basic cardiac function and morphology.

	CPT group	EH group	*P* value
LV EDWT (mm)*	10.5 ± 1.6	12.4 ± 2.8	**0.03**
LV ESWT (mm)	13.8 ± 2.8	15.7 ± 2.5	0.053
LV EF (%)	58.3 ± 7.6	57.6 ± 7.9	0.82
LV EDVI (mL/m^2^)	77.0 ± 9.0	69.8 ± 15.3	0.11
LV ESVI (mL/m^2^)	33.7 (24.3–38.1)	34.6 (24.8–36.5)	0.78
LV SI (mL/m^2^)	44.9 ± 6.6	41.2 ± 7.7	0.16
LV CI (mL/min/m^2^)	3109 (2776–3672)	2753 (2573–3091)	0.07
LV MI (g/m^2^)	49.0 ± 13.2	57.9 ± 17.7	0.12

CI, cardiac index; CPT, catecholamine-producing tumor; EDVI, end-diastolic volume index; EDWT, end-diastolic wall thickness; EF, ejection fraction; EH, essential hypertension; ESVI, end-systolic volume index; ESWT, end-systolic wall thickness; LV, left ventricle; MI, mass index; SI, systolic volume index. Bold indicates statistical significance.

### LV myocardial strain

Global strains of the three directions and the systolic and diastolic peak strain rates demonstrated no significant differences between the two groups. In segmental strain analysis, the CPT group demonstrated significantly higher circumferential strain of the basal wall than the EH group (19.4 ± 3.2% vs 16.8 ± 3.6%; *P* = 0.04); however, apical circumferential strain was not significantly different (25.0 ± 6% vs 22.6 ± 5.6%; *P* = 0.25). There was also no significant difference in the radial and longitudinal strains in the apical and basal walls between the two groups (Table 3).

**Table 3:** Global and regional myocardial strain.

	CPT group	EH group	*P* value
Global strain			
Peak strain (%)			
Radial	62.5 ± 15.0	64.0 ± 26.8	0.86
Circumferential	−20.2 ± 3.0	−18.2 ± 3.9	0.11
Longitudinal	−24.7 ± 3.8	−24.3 ± 4.7	0.78
Peak systolic strain rate (s^−1^)			
Radial	3.4 ± 1.0	3.7 ± 1.7	0.58
Circumferential	−1.2 ± 0.2	−1.1 ± 0.3	0.29
Longitudinal	−1.5 ± 0.3	−1.4 ± 0.4	0.67
Peak diastolic strain rate (s^−1^)			
Radial	−4.1 ± 1.2	−3.4 ± 1.5	0.21
Circumferential	1.1 ± 0.3	1.0 ± 0.4	0.5
Longitudinal	1.3 (1.0–1.6)	1.1 (1.0–1.8)	0.56
Regional strain			
Basal peak strain (%)			
Radial	66.9 ± 12.9	65.7 ± 17.2	0.82
Circumferential*	−19.4 ± 3.2	−16.8 ± 3.7	**0.04**
Longitudinal	−24.8 ± 3.8	−24.3 ± 5.0	0.76
Apical peak strain (%)			
Radial	55.8 ± 20.2	68.4 ± 31.7	0.19
Circumferential	−25.0 ± 6.1	−22.6 ± 5.6	0.25
Longitudinal	−24.5 ± 4.0	−24.3 ± 4.6	0.91

CPT, catecholamine-producing tumor; EH, essential hypertension. Bold indicates statistical significance.

### Native T1 values

**Figure 2:**
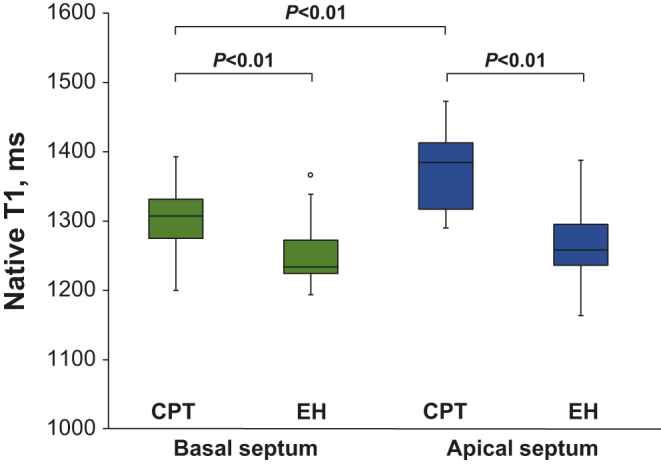
Box plots showing native T1 values in the basal and apical septal walls of the CPT and EH groups. Box plots show the medians, quartiles, ranges and outliers. Comparison between the CPT and EH groups was performed using Student’s *t*-test, and between the basal and apical septum in the same group using the paired *t*-test. Native T1 values in the CPT group were significantly higher than those in the EH group in both the basal septum and apical septum. In the CPT group, native T1 values in the apical septum appeared significantly higher than those in the basal septum; however, there were no significant differences in the values between the apical septum and basal septum in the EH group. * indicates statistical significance.

**Figure 3:**
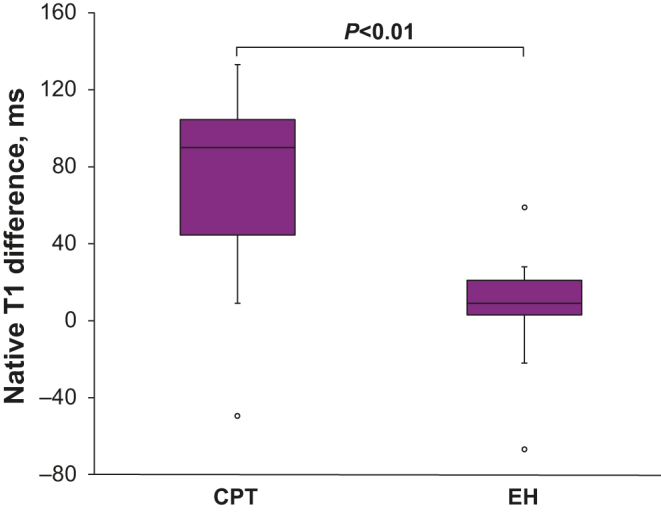
Box plots showing the difference in native T1 values between the apical septum and basal septum (basal values subtracted from apical values) in the CPT and EH groups. The difference in the CPT group was significantly greater than that in the EH group. * indicates statistical significance.

The ICCs of the T1 values measured in all the patients were 0.93 (95% CI: 0.85–0.96) in the basal segments and 0.99 (95% CI: 0.989–0.997) in the apical segments, indicating excellent reliability. Patients with CPT demonstrated significantly higher native T1 values than those with EH, in the mid-wall of both the basal septum (1307 ± 48 ms vs 1241 ± 45 ms; *P* < 0.01) and the apical septum (1377 ± 59 ms vs 1265 ± 58 ms; *P* < 0.01). In the CPT group, native T1 values on the mid-wall of the apical septum were significantly higher than those on the basal septum (1377 ± 59 ms vs 1308 ± 49 ms; *P* < 0.01); however, the EH group did not demonstrate significant differences in native T1 values between the apical and basal walls (1265 ± 58 ms vs 1258 ± 48 ms; *P* = 0.34) (Fig. 2). The differences in native T1 values in the apical and basal interventricular septum were significantly higher in the CPT group than in the EH group (69 ± 47 ms vs 10 ± 29 ms; *P* < 0.01) (Fig. 3).

### Correlation between MR parameters and biochemical phenotype

Among 16 CPT patients, eight were the adrenergic type and eight were the noradrenergic type. The MR parameters were not significantly different between the two groups.

## Discussion

In this 3T CMR study of patients with CPT, we showed that the native T1 values in patients with CPT were significantly higher than those in patients with EH. Additionally, the apical septum’s native T1 values were significantly higher than those of the basal septum in the CPT group, whereas in the EH group, there were no significant differences between the apical and basal septum’s native T1 values. Furthermore, patients with CPT demonstrated significantly higher basal circumferential strain than those with EH, despite comparable LV global function.

Ferreira *et al.* have reported a systemic study of catecholamine-induced cardiac injury using a 1.5T MR scanner (18). They showed that patients with pheochromocytoma presented with lower LVEF, peak systolic circumferential strain and diastolic circumferential strain rate and higher native T1 values than EH patients. Our results were in line with the findings of Ferreira *et al.* in terms of the higher myocardial native T1 values in the CPT group than in the EH group. Another report indicated elevated native T1 values in severely hypertensive patients with LV hypertrophy compared to those in normotensive control subjects (24). However, in our study, there were no significant differences in the basic cardiac function between the two groups. The discrepancy between the studies may be due to the different study populations; our study may have enrolled patients with less severe cardiac damage than the study by Ferreira *et al.* Nonetheless, taken together, the results of the previous and present study indicate that T1-mapping can be a more sensitive tool than wall motion assessment for detecting catecholamine-induced cardiac injury.

Pathologically, catecholamines have been shown to cause myocardial injury, such as contraction band necrosis, via alpha-receptor-mediated vasoconstriction and oxygen-derived free radical injury, leading to myocyte membrane damage and death (25, 26). Moreover, autopsies of patients who died due to pheochromocytoma showed cardiac lesions, such as focal degeneration and necrosis of myocardial fibers, foci of inflammatory cells and diffuse myocardial edema, which were designated as catecholamine-induced myocarditis (26). The elevation of the native T1 values in our study may reflect the early stages of myocardial damage, with preserved cardiac function, in patients with CPT.

Although our study demonstrated no significant differences in global cardiac function or morphology between the CPT and EH groups, except for end-diastolic wall thickness, the LVEF of the patients in both groups were toward the lower limit of normal values as compared with the reported normal values obtained with CMR (27). Our subjects might have had a similar extent of mildly impaired LVEF, without a significant difference between the groups.

In terms of global strain analysis, the two groups in our study demonstrated no significant differences, but both groups might also have had impaired myocardial global circumferential strain as compared with the reported normal values for myocardial strain (28, 29). Previous studies have reported difficulty in matching hypertensive control subjects, because catecholamine-induced hypertension is highly variable and often paroxysmal, occurring on a background of sustained hypertension or presenting as normotension in between periods of hypertension (6, 30). Our patients in the control EH group had been taking more than two types of antihypertensive drugs for a significantly longer time than those in the CPT group and were referred for the work-up of hypertension; therefore, there might have been a selection bias in the control group, because mildly hypertensive patients might not have been referred to our endocrinology specialists. Previous reports have demonstrated a relationship of increased wall thickness with impaired myocardial strain (31, 32); our circumferential strain results in the EH group may be associated with a thickened myocardial wall.

Previous reports have shown that plasma catecholamine levels are markedly higher among patients with Takotsubo (stress-induced) cardiomyopathy (33), and mammalian hearts have a higher concentration of β-adrenoceptors in the apical myocardium, with the concentration decreasing in a gradient from the apex to the base (19, 34). These findings could explain the regional differences in response to high catecholamine levels, with circulating epinephrine having a greater influence on apical, relative to basal, function (20). The regional distribution of β-adrenoceptors might have also contributed to the significantly higher native T1 values in the apical wall and the relatively preserved circumferential strain in the basal wall in the CPT group. Our results may indicate that the apical wall may be prone to catecholamine-induced injury.

A previous study showed that native T1 values in patients with pheochromocytoma decreased after surgical resection along with LV mass regression, but did not completely normalize (18). Follow-up MR studies are needed to reveal whether resection of tumors improves cardiac function, elevated native T1 values and the difference in strain and native T1 values between the apical and basal septum. Moreover, clinical follow-up is also needed to determine the long-term clinical significance of these results.

This study had several limitations. First, although we demonstrated regional variance of T1 values in CPT patients, the sample size was relatively small. A larger study is warranted to confirm our results. Second, native T1 values are increased by several mechanisms, such as cardiomyocyte damage, enlarged extracellular space, intramyocardial edema and fibrosis (17). We did not acquire late gadolinium enhancement (LGE) images and post-contrast T1-mapping to evaluate the extracellular volume fraction. A previous systemic MR study in pheochromocytoma patients showed that 58% of patients presented with disseminated focal myocardial lesion pathologically, and 59% of these patients had a nonischemic pattern on LGE (18, 26). Therefore, we might have missed focal myocardial lesions, such as subclinical myocardial infarction. Such focal lesions might have contributed to the elevated native T1 values in the apical region. Nonetheless, native T1 values might comprehensively reflect several mechanisms underlying catecholamine-induced cardiac injury. Finally, endomyocardial biopsy was not performed in these patients, and thus direct histopathological correlation with the imaging findings was not possible.

In conclusion, T1-mapping using a 3T MR scanner is a sensitive tool for detecting subclinical catecholamine-induced cardiac injury and the influence of catecholamines on myocardial tissue, such as myocardial edema or fibrosis. We demonstrated that the injury may be greater in the apical than in the basal septum. Larger sample sizes and follow-up studies are needed to determine whether these subclinical findings are related to clinical prognosis and whether early intervention can minimize the catecholamine-induced myocardial injury.

## Supplementary Material

Supplementary Table: Magnetic resonance scan parameters

## Declaration of interest

The authors declare that there is no conflict of interest that could be perceived as prejudicing the impartiality of the research reported.

## Funding

This research did not receive any specific grant from any funding agency in the public, commercial or not-for-profit sector.
