# Supplementary material for: 3T MRI evaluation of regional catecholamine-producing tumor-induced myocardial injury
Source: Endocr Connect. 2019 Mar 25;8(5):454–61. doi: 10.1530/EC-18-0553 (PMC6479192; doi:10.1530/EC-18-0553)
Supplement: Supplementary Table: Magnetic resonance scan parameters [file supplementary_table_1.pdf]

Supplementary Table: Magnetic resonance scan parameters

Parameters	**Cine**	**T1-mapping**
Scan sequence	Balanced steady-state free precession	Modified Look-Locker inversion recovery
Repetition time/echo time, ms	60.75/1.08	600-750/0.82–1.06
Flip angle, °	40-43	35
In-plane pixel size, mm	1.3 × 1.3	1.3-1.8 × 1.3–1.8
Slice thickness, mm	8	8
Parallel imaging, acceleration factor	2	2
Band width, Hz/Px	1302	930–1302
Sampling schema for T1 mapping	n.a.	8(2)2*

*8(2)2 protocol indicates that there were a total of 2 inversions; 8 images were acquired over 8 inter-beat (RR) intervals of electrocardiogram after the first inversion, followed by a waiting period of 2 RR intervals, and then 2 images were acquired after the second inversion.
